# Expediting Next-Generation Hybrid Technology in Recalcitrant Maize Inbreds through In Vivo Targeted Activity of CRISPR/Cas9

**DOI:** 10.3390/ijms25115832

**Published:** 2024-05-27

**Authors:** Liudi Hou, Bing Xiao, Jinjie Zhu, Changlin Liu, Qingyu Wu, Chuanxiao Xie, Huawen Zou, Xiantao Qi

**Affiliations:** 1 College of Agriculture, Yangtze University, Jingzhou 434000, China; houliudi@126.com; 2 Institute of Crop Science, Chinese Academy of Agricultural Sciences, Beijing 100081, China; zhujinjie@caas.cn (J.Z.); liuchanglin@caas.cn (C.L.); xiechuanxiao@caas.cn (C.X.); 3 State Key Laboratory of Efficient Utilization of Arid and Semi-arid Arable Land in Northern China, Institute of Agricultural Resources and Regional Planning, Chinese Academy of Agricultural Sciences, Beijing 100081, China; 17319374552@163.com (B.X.); wuqingyu@caas.cn (Q.W.); 4 College of Resources and Environmental Sciences, National Academy of Agriculture Green Development, Key Laboratory of Plant-Soil Interactions, Ministry of Education, China Agricultural University, Beijing 100193, China

**Keywords:** *Zea mays* L., molecular breeding, next-generation hybrid technology, CRISPR/Cas9, genic male sterile

## Abstract

The Manipulated Genic Male Sterile Maintainer (MGM) system, a next-generation hybrid seed technology, enables efficient production of sortable seeds from genic male sterile (GMS) lines. However, implementing robust MGM systems in commercial maize inbred lines requires stable transformation, a genotype-specific and laborious process. This study aimed to integrate MGM technology into the commercial maize inbred line Z372, developing both GMS and MGM lines. We utilized the MGM line ZC01-3A-7, which contains the MS26ΔE5 editor T-DNA and MGM T-DNA, previously established in the highly transformable ZC01 recipient plants. Through a combination of crossing and backcrossing with Z372, we targeted the fertility gene *Ms26* within the Z372 genome for mutation using the in vivo CRISPR/Cas9 activity within the MS26ΔE5 editor T-DNA construct. This approach facilitated precise editing of the *Ms26* locus, minimizing linkage drag associated with the *Ms26* mutation. Whole-genome SNP analysis achieved a 98.74% recovery rate for GMS and 96.32% for MGM in the BC2F2 generation. Importantly, the Z372-GMS line with the *ms26ΔE5* mutation is non-transgenic, avoiding linkage drag and demonstrating production readiness. This study represents a significant advancement in maize breeding, enabling the rapid generation of GMS and MGM lines for efficient hybrid seed production.

## 1. Introduction

Maize (*Zea mays* L.) is one of the world’s most important cereal crops, contributing significantly to global food security. Its widespread cultivation is further enhanced by its remarkable ability to exploit heterosis, a phenomenon that boosts yield and vigor in hybrid varieties [1]. The utilization of male sterility is a key component in ensuring the purity and efficiency of hybrid seed production, which is fundamental for maximizing maize yields and genetic gain. Male sterility in maize is broadly classified into two primary types based on the source of fertility genes: cytoplasmic male sterility (CMS) and genic male sterility (GMS) [2]. Cytoplasmic male sterility (CMS) has played a central role in the development and success of the three-line seed production technology, which has revolutionized maize hybrid seed production [3]. Furthermore, the widespread utilization of the three-line seed production technology has been constrained by the limited availability of suitable parental lines, which restricts the genetic diversity available for breeding. This, in turn, can lead to unreliable restoration of CMS lines, a consequence of the complex interactions between cytoplasmic and nuclear genomes [4]. Genic male sterility (GMS), which relies on recessive nuclear sterility genes, has faced significant barriers to its commercial application, primarily due to the challenges in effectively segregating sterility lines from maintainer lines. However, photoperiod- and/or thermosensitive genic male sterility (PTGMS) offers a unique solution, as the same line can serve as both the male sterility line and the maintainer line, with its function determined by environmental conditions [5]. The versatility of PTGMS, while offering valuable opportunities for breeding programs, introduces complexities in managing diverse traits within the same genetic background. The advent of seed production technology (SPT) has significantly advanced the field of maize hybrid seed production by overcoming the challenges associated with genic male sterility. By enabling distinct identification of male sterility and maintainer lines, SPT has not only expanded production methods but also unlocked its full potential [6]. Moreover, the development of multi-control sterility technology has further broadened its applications, leading to significant improvements in the efficiency of maize hybrid seed production [7]. However, the successful integration of SPT technology relies on the creation and incorporation of GMS genes. Traditional breeding methods, whether through natural mutation or artificial mutagenesis, face limitations such as lengthy breeding cycles and reduced efficiency. Additionally, linkage drag during backcross breeding further hinders the widespread adoption of this approach [8]. These challenges necessitate innovative strategies to overcome these hurdles and facilitate the seamless implementation of SPT technology.

Gene editing technologies, particularly CRISPR/Cas9 [9], have revolutionized genetic modification, enabling targeted and precise genetic enhancements in maize [10,11,12,13]. Building upon our previous work [14], we have developed the Manipulated GMS Maintainer (MGM) system, a next-generation hybrid seed technology that integrates CRISPR/Cas9 technology with SPT. This innovation addresses the limitations of the SPT system by facilitating the efficient creation of sterile genes. The MGM technology consists of two primary components. The first is the MS26ΔE5 editor T-DNA, a CRISPR/Cas9 vector engineered to precisely edit the fourth intron and 3′ untranslated region (UTR) of the maize male fertility gene *MS26*, resulting in the deletion of the fifth exon and the creation of the *ms26ΔE5* mutation. The second component is the MGM T-DNA vector, which includes three expression cassettes: (1) one driven by the native *Ms26* promoter to express the *Ms26* coding sequence (CDS), preventing gene editing by MS26ΔE5 editor and restoring fertility to *ms26ΔE5* mutants; (2) a pollen-specific promoter PG47 to express the *ZmAA1* gene, inducing the death of pollen carrying the MGM vector; and (3) an endosperm-specific promoter *LTP2* to express *DsRed* for the selection of maintainer lines carrying the MGM vector. ZC01-MGM plants were developed by co-transforming the MS26ΔE5 editor T-DNA and MGM T-DNA into the highly transformable maize inbred line ZC01, utilizing the Agrobacterium-mediated transformation of immature embryos. In ZC01-MGM plants, the fifth exon of *MS26* was precisely deleted by the MS26ΔE5 editor, creating a *ms26ΔE5* GMS mutant. Fertility was restored in half of the male gametes by MGM, while the other half remained sterile due to induced starch degradation. The inclusion of a fluorescent protein in MGM facilitated the straightforward identification of hemizygous seeds. This system enabled the non-invasive sorting of maintainer and sterile seeds, thereby streamlining efficient hybrid seed production. The sorted GMS sterile line was deployed in hybrid seed production, while the MGM maintainer line seeds were preserved for subsequent propagation, establishing an exceptionally efficient next-generation hybrid seed production technology. This technique not only efficiently produced hybrid seeds but also preserved sterility in the line, signifying a paradigm shift in next-generation hybrid seed production technology. However, the implementation of MGM technology for the development of commercial inbred lines typically relies on transgenic methods [10,11,12,13]. Furthermore, achieving stable transformation with plant regeneration in maize is genotype-specific and often requires laborious and time-consuming tissue culture procedures [15]. Therefore, for staple crop species such as maize, a practical approach involves the stable transformation of selected genotypes that are amenable to the process, leveraging their endogenous genome-editing machinery in trans mode to introduce the desired mutations [8,16].

The objective of this study was to enhance the efficiency of next-generation hybrid seed production technology in commercial maize inbred lines. We aimed to utilize the MGM line ZC01-3A-7, previously established in the readily transformable inbred line ZC01, for backcross breeding with the commercial inbred line Z372. Our strategy involved the use of the CRISPR/Cas9 system present in ZC01-3A-7 to precisely target and delete the *Ms26* gene within the Z372 genome. This approach was designed to generate a mutation type, *ms26ΔE5*, while avoiding the introduction of linkage drag associated with the *ms26ΔE5* mutation. Concurrently, we employed whole-genome SNP analysis to identify and select for Z372-MGM and Z372-GMS lines with a high degree of genetic background restoration. By integrating these advanced techniques, we have not only effectively addressed the challenges of establishing MGM technology in previously recalcitrant recipient materials but also sought to significantly enhance the breeding efficiency of commercial inbred lines, thereby paving the way for the next-generation hybrid seed production.

## 2. Results

### 2.1. The Experimental Strategy and Rationale of the Design

**Figure 1:**
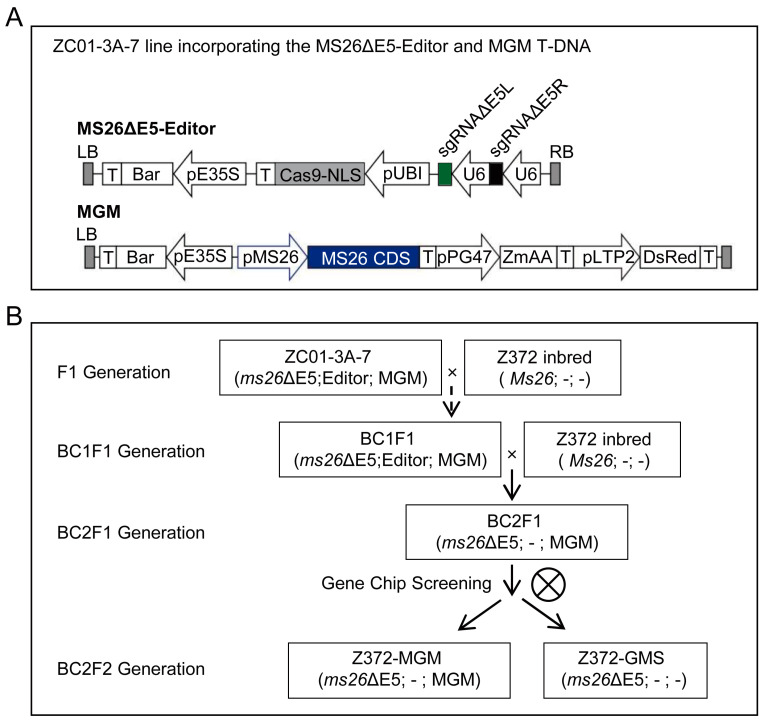
Schematic overview of the efficient creation of Z372 GMS and MGM lines with genetic background restoration. (**A**) The panel depicts the ZC01-3A-7 donor line, which carries both the MS26ΔE5 editor T-DNA and MGM T-DNA constructs. (**B**) The rational design and experimental strategy underlying the efficient generation of Z372 GMS and MGM lines, with a focus on genetic background restoration.

To efficiently derive GMS and MGM lines from the target inbred line Z372, we implemented a breeding strategy integrating backcrossing with whole-genome SNP screening (Figure 1). The line ZC01-3A-7, which harbors both the MS26ΔE5 editor T-DNA and the MGM T-DNA, was selected as the maternal line (Figure 1A) [14]. The inbred Z372, originating from a single cross, was chosen as the paternal line. Crossing ZC01-3A-7 with Z372, followed by repeated backcrossing with Z372 as the recurrent parent, resulted in the production of the BC2F2 generation. From the F1 to BC1F1 generations, plants exhibiting red fluorescent kernels and carrying the MS26ΔE5 editor T-DNA were selected for crossing with Z372 to produce the BC1F1 generation. Molecular testing was conducted from BC1F1 to BC2F1 to ensure the fulfillment of three criteria: (1) the presence of red fluorescent grains, (2) the presence of MS26ΔE5 editor T-DNA, and (3) the absence of the *ms26ΔE5* mutation genotype from ZC01-3A-7. Individuals meeting these conditions were backcrossed with Z372 to generate the BC2F1 line. From BC2F1 to BC2F2, plants exhibiting red fluorescent grains and harboring the *ms26ΔE5* mutation were selected for self-pollination to produce the BC2F2 generation. Within the BC2F2 population, plants exhibiting red fluorescent kernels that were homozygous for the *ms26ΔE5* mutation and confirmed to lack the MS26ΔE5 editor T-DNA were identified as candidate Z372-MGM plants. Conversely, plants with non-red fluorescent kernels, also homozygous for the *ms26ΔE5* mutation and confirmed to be devoid of the MS26ΔE5 editor T-DNA, were identified as candidate Z372-GMS plants. Both candidate Z372-MGM and Z372-GMS plants underwent whole-genome SNP analysis. Those with the highest genetic background recovery rate to Z372 and containing the MGM T-DNA were selected to constitute the Z372-MGM line, whereas those without the MGM T-DNA were designated to form the Z372-GMS line (Figure 1B).

### 2.2. Generation and Characterization of the ms26ΔE5 Mutant in Z372 Inbred Line

To prevent the introduction of linkage drag from backcrossing the *ms26ΔE5* mutant allele into the Z372 progeny, we employed CRISPR/Cas9-mediated gene editing to precisely target the *MS26* locus in Z372. This strategy allowed us to generate novel *ms26ΔE5* mutants within the Z372 genetic background. In the BC1F1 generation, PCR screening of 93 individual BC1F1 plants identified 23 plants that lacked the *ms26ΔE5* mutant allele from ZC01-3A-7 while harboring the MS26ΔE5 editor T-DNA. These plants were selected as the maternal lines for backcrossing with Z372 (Supplementary Table S1). In the BC2F1 generation, screening of 94 individuals revealed that 4 plants had acquired a novel *ms26ΔE5* mutant allele through CRISPR/Cas9 editing, resulting in a gene knockout efficiency of 4.26% (4/94) (Supplementary Table S1). Self-pollination of the four selected BC2F1 plants produced 20 BC2F2 individuals homozygous for the *ms26ΔE5* mutation (Table 1, Supplementary Table S2) and devoid of the MS26ΔE5 editor T-DNA (Supplementary Table S1). Among these 20 plants, those lacking the MGM T-DNA were designated as candidate Z372-GMS plants, named Z372-GMS1 to Z372-GMS10, and were subsequently subjected to whole-genome SNP analysis.

### 2.3. Generation and Characterization of the MGM Line in Z372

To obtain the MGM line within the Z372 inbred line, a backcross breeding strategy was employed to integrate the MGM T-DNA from the ZC01-3A-7 line into the Z372 genome through genetic recombination. The presence of an observable red fluorescent kernel marker within the MGM T-DNA (Figure 1A) allowed for the identification of kernels harboring the MGM T-DNA, which exhibited red fluorescence upon excitation with 550 nm light, while kernels lacking the MGM T-DNA did not emit red fluorescence under the same conditions. Utilizing this kernel marker, kernels exhibiting red fluorescence were selected for backcrossing from the F1 to BC2F1 generations. In the BC2F2 generation, plants meeting three criteria were chosen: (1) containing the MGM T-DNA, (2) being homozygous for the *ms26ΔE5* mutation, and (3) lacking the MS26ΔE5 editor T-DNA. Molecular screening of the BC2F2 population identified 10 individual plants conforming to these criteria, designated as candidate Z372-MGM plants, numbered Z372-MGM1 to Z372-MGM10, and subsequently subjected to whole-genome SNP analysis (Supplementary Table S1, Table 1).

**Figure 2:**
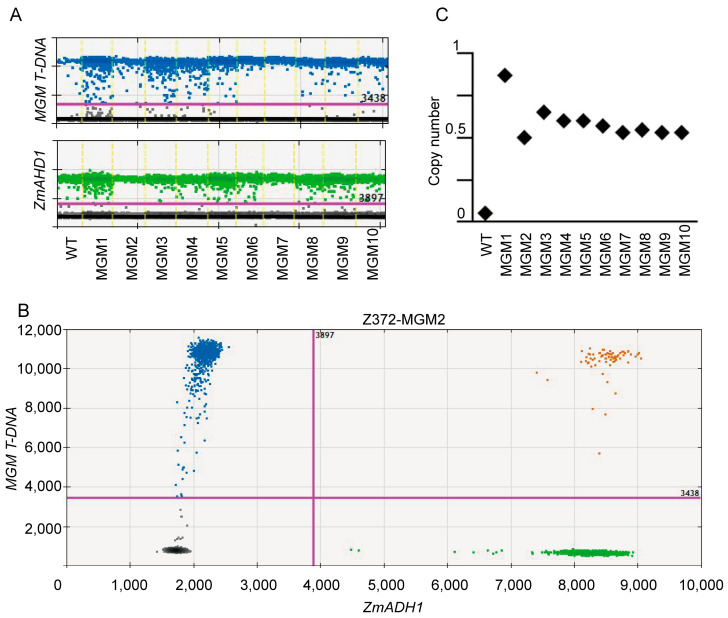
Assessment of MGM T-DNA copy number in Z372-MGM candidate material using ddPCR technology. (**A**) Represents the 1D droplet digital PCR (ddPCR) plot displaying the count of MGMT-DNA-positive droplets (blue droplets and orange droplets) and *ZmADH1* gene droplets (green droplets and orange droplets) for ten candidate Z372-MGM plants. (**B**) Presents the detailed 2D ddPCR data for a single plant, Z372-MGM2. (**C**) Depicts the calculated MGM T-DNA copy numbers for plants Z372-MGM1 to Z372-MGM10.

Furthermore, the MGM T-DNA contains a pollen-killer element, which prevents pollen carrying the MGM T-DNA from being transmitted to the next generation via male gametes. Therefore, to ensure the proper functioning of the MGM system, the MGM T-DNA must be present in a single-copy hemizygous state in the candidate plants. This configuration allows for the *ms26ΔE5* mutation to be propagated through male gametes to produce GMS in the progeny (Figure 1A). To ascertain the copy number of the MGM T-DNA in the candidate Z372-MGM plants, digital droplet PCR technology was employed, using the maize single-copy gene *ZmADH1* as an internal reference and sequences on the MGM T-DNA vector as targets for detection. The experimental results indicated that the MGM T-DNA in plants Z372-MGM2 to Z372-MGM10 was present in a single-copy hemizygous state (Figure 2, Supplementary Table S3).

### 2.4. Whole-Genome SNP Analysis and Selection of Z372-GMS and Z372-MGM Plants

To achieve GMS and MGM lines identical to the Z372 genetic background, all ten Z372-GMS candidate plants (Z372-GMS1 to Z372-GMS10) and the corresponding ten Z372-MGM candidate plants were subjected to whole-genome SNP detection using the maize 45 K liquid chip, with the genetic background restoration rate of the screened materials being calculated. The SNPs measured were filtered based on three criteria: (1) parental genotype filtering, which excluded loci with missing parental information and selected markers where both parents were homozygous and polymorphic between them; (2) completeness filtering, which included markers genotyped in more than 80% of all progeny; and (3) target region representative SNP marker selection, where for each probe-covered region, the SNPs with the lowest missing proportion were extracted as the representative SNP markers for that target region. The total SNPs meeting these conditions were then analyzed for background restoration rate. After data filtering, each sample yielded approximately 25.7 K valid SNPs. Utilizing valid SNPs, the number of SNPs unique to Z372, as well as those unique to ZC01, and the heterozygous sites containing SNPs from both Z372 and ZC01 were calculated, from which the genetic background restoration rate of the samples was derived. Among the Z372-MGM candidate plants, the genetic background restoration rate ranged from 93.82% to 96.32%, with the highest rates observed in Z372-MGM2 and Z372-MGM7 at 96.32%. In the Z372-GMS candidate plants, the background restoration rate varied from 95.06% to 98.74%, with Z372-GMS8 exhibiting the highest restoration rate (Table 2).

### 2.5. Phenotypic Evaluation and Stability Assessment of Z372-GMS

**Figure 3:**
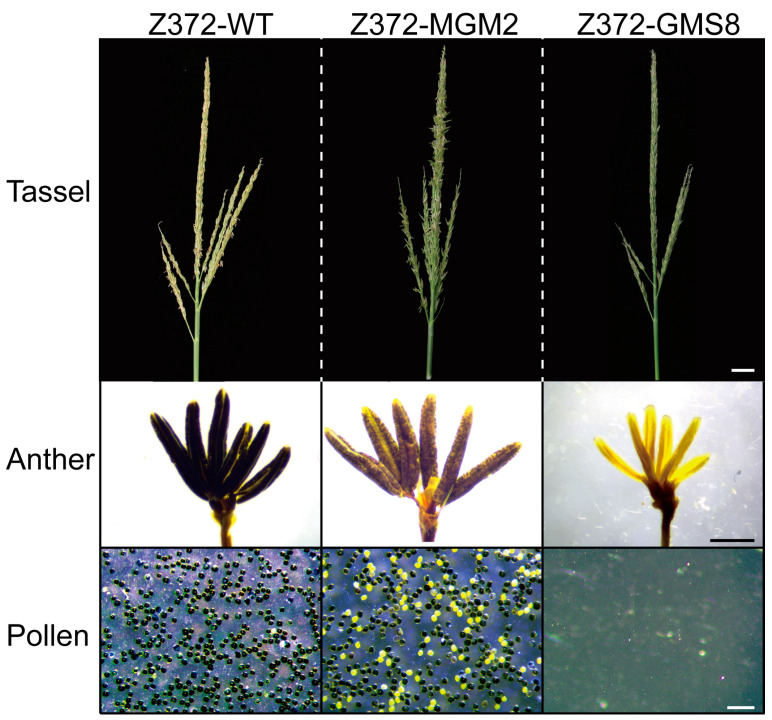
Phenotypic analysis of fertility traits in Z372, Z372-MGM, and Z372-GMS lines. The upper panel represents the tassels of the plants, the middle panel displays the anthers, and the lower panel showcases the pollen. The panels on the left depict the inbred line Z372, while those on the right illustrate the Z372-GMS8. The panels situated between Z372 and Z372-GMS8 correspond to the Z372-MGM2 line.

To assess the Z372-GMS phenotype, plant Z372-GMS8 was selected as the experimental material for phenotypic evaluation. This plant was homozygous for the *ms26ΔE5* mutation and exhibited the highest rate of genetic background restoration. Observation of the Z372-GMS8 tassel revealed that the anthers were not exposed during the flowering period. Microscopic observation confirmed that the anthers were withered. Dissection of Z372-GMS8 anthers showed an absence of pollen grains, indicating complete male sterility (Figure 3). These findings demonstrate that the breeding strategy employed can successfully yield a phenotypically stable GMS line with a Z372 genetic background as early as the BC2F2 generation.

### 2.6. Phenotypic and Molecular Characterization of Z372-MGM

To further assess the phenotype of the Z372-MGM plant, Z372-MGM2, which contained the MGM T-DNA, lacked the MS26ΔE5 editor T-DNA, was homozygous for the *ms26ΔE5* mutation, and exhibited the highest rate of genetic background restoration, was selected for study. Due to the presence of the complete *MS26* gene CDS within the MGM T-DNA (Figure 1A), the Z372-MGM2 plant is capable of restoring the male sterility phenotype resulting from being homozygous for the *ms26ΔE5* mutation. Observation of the tassel during the flowering stage revealed anther extrusion, and examination of the anther confirmed the presence of viable pollen grains within the Z372-MGM2 anther (Figure 3). However, with the MGM T-DNA present as a single-copy hemizygous in Z372-MGM2, half of the pollen contains the MGM T-DNA, while the other half lacks it. Owing to the pollen suicide gene within the MGM T-DNA, observation of the Z372-MGM2 anther revealed a 1:1 ratio of viable to non-viable pollen, with half of the pollen being active and the other half being dead (Figure 3 and Table 3).

### 2.7. Assessment of Z372-MGM’s Ability to Propagate Z372-GMS

**Figure 4:**
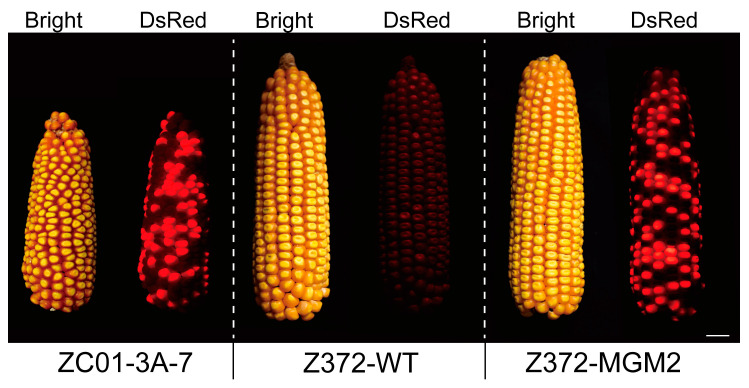
Ear phenotypes of ZC01-3A-7, Z372 inbred line, and Z372-MGM2 under bright and 550 nm excitation light. The left panel displays the ear phenotype of ZC01-3A-7 under bright and 550 nm excitation light, the central panel shows the ear phenotype of the Z372 inbred line under the same lighting conditions, and the right panel illustrates the ear phenotype of Z372-MGM2 under bright and 550 nm excitation light.

To evaluate Z372-MGM’s ability to propagate Z372-GMS lines, the Z372-MGM2 plant was selected for investigation. Since the Z372-MGM2 plant is homozygous for the *ms26ΔE5* mutation, the fertile pollen of Z372-MGM2 carries the genotype containing the *ms26ΔE5* mutation. In the Z372-MGM2 plant, the MGM T-DNA and its absence are distributed in a 1:1 ratio across different kernels within an ear, resulting in a 1:1 segregation of MGM to GMS kernels upon self-pollination. Consequently, kernels expressing the selection marker *DsRed* in the ear represent the GMS maintainer line (MGM), while kernels that do not fluoresce red represent the GMS sterile line, utilized for hybrid seed production (Figure 4).

### 2.8. Agronomic Trait Assessment of Z372-GMS and Z372-MGM Plants

**Figure 5:**
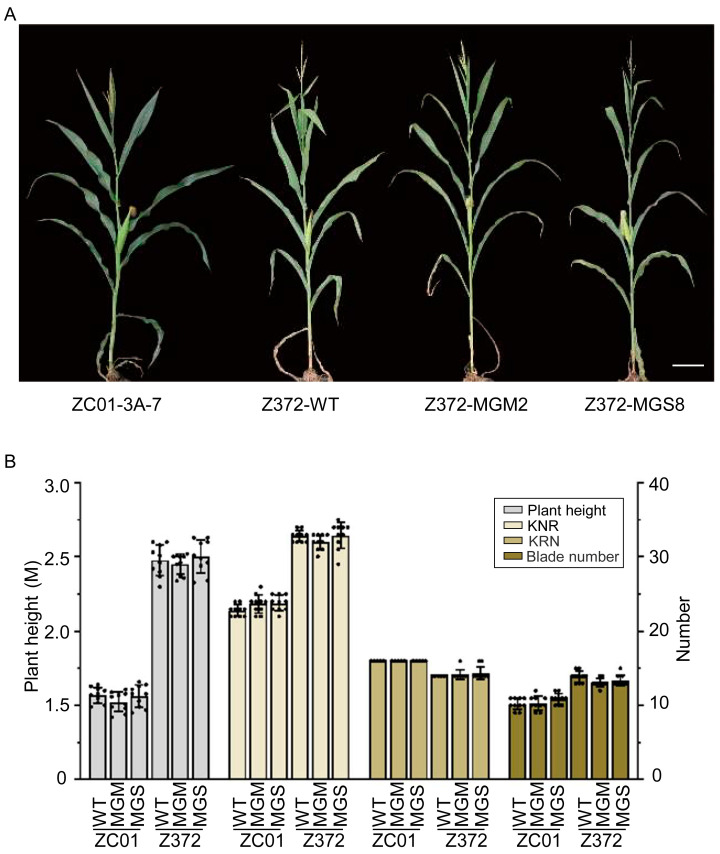
Plant phenotype and agronomic traits of Z372-MGM and GMS lines. (**A**) Phenotypes of ZC01-3A-7, Z372, Z372-MGM, and Z372-GMS plants, bar = 20 cm. (**B**) Statistical analysis of plant height, kernel row number (KRN), kernel number per row (KNR), and blade number in ZC01-3A-7, Z372, Z372-MGM, and Z372-GMS materials.

To evaluate the agronomic traits of Z372-GMS and Z372-MGM plants, we assessed various traits, including plant height, leaf number, kernel row number (KRN), and kernel number per row (KNR) (Figure 5). Phenotypic observations revealed that the Z372-MGM and Z372-GMS plants, derived from this breeding strategy, were consistent in plant morphology with the Z372 inbred line and showed significant differences from ZC01-3A-7 (Figure 5A). Plant height, leaf number, KRN, and KNR were measured for ZC01, Z372, and their corresponding MGM and GMS lines. The experimental data indicated that Z372-MGM and Z372-GMS were phenotypically consistent with the Z372 inbred line (Figure 5B). These data collectively demonstrate that the technology employed in this study constitutes an efficient and reliable breeding strategy for the development of GMS and MGM maintainer lines in previously recalcitrant inbred lines.

## 3. Discussion

The development of efficient and reliable methods for generating genic male sterility (GMS) and maintainer lines (MGM) in maize is crucial for advancing hybrid seed production. While traditional breeding methods can be time-consuming and often lead to linkage drag, recent advances in gene editing technology, particularly CRISPR/Cas9, offer a promising avenue for streamlined GMS line development. However, the successful implementation of gene editing in commercial maize varieties often requires genotype-specific approaches, which can be challenging and labor-intensive. This study demonstrates a highly efficient strategy for integrating next-generation hybrid seed technology, specifically the MGM system, into the commercial maize inbred line Z372. We achieved this by combining precise CRISPR/Cas9-mediated gene editing with a well-defined backcross breeding strategy. Our approach targeted the fertility gene *Ms26* within Z372, resulting in the generation of GMS lines with a high background recovery rate, minimizing linkage drag. This approach is particularly advantageous as it eliminates the need for transgene integration, which is often associated with regulatory hurdles and public perception concerns.

### 3.1. The ms26ΔE5 Mutation in Z372 MGM Was Introduced through Genetic Editing, Rather than Resulting from Genetic Introgression

Previous research from our team has demonstrated that stable CRISPR/Cas9 expression can achieve a mutation rate of approximately 20% [8,16]. In this study, we utilized ZC01-3A-7 as the CRISPR/Cas9 donor plant, leveraging its endogenous activity to introduce mutations into the *ms26ΔE5* gene of Z372. To prevent the introgression of the ZC01-3A-7 *ms26ΔE5* mutation into Z372, we employed a stringent triple selection process spanning the BC1F1 to BC2F1 generations. This selection involved screening for the absence of the ZC01-3A-7 *ms26ΔE5* mutation, the presence of CRISPR/Cas9, and the presence of the MGM cassette. Notably, genotype analysis of BC2F2 plants revealed no evidence of the ZC01-3A-7 *ms26ΔE5* mutation (Supplementary Table S2). These findings strongly suggest that the *ms26ΔE5* mutation in Z372 MGM arose exclusively through CRISPR/Cas9 editing of the Z372 genome rather than through genetic introgression from the donor plant.

### 3.2. The Linked Drag Introduced by MGM Cannot Be Transmitted to Z372 GMS

**Figure 6:**
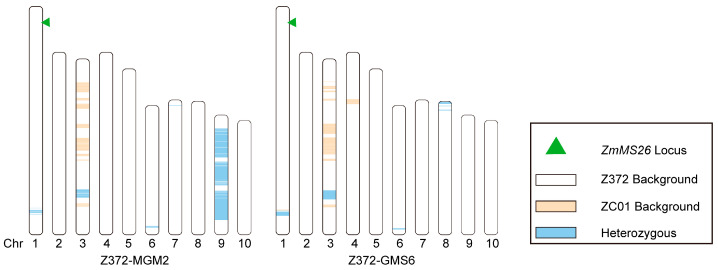
Genome background recoveries of Z372-MGM2 and Z372-GMS6.

Our previous research [14] confirmed that the ZC01-3A-7 strain was a transgenic plant containing two T-DNAs. During the breeding process for Z372-MGM, a significant event occurred: the T-DNA encoding CRISPR/Cas9 was excised, while the remaining MGM T-DNA was seamlessly integrated into the Z372-MGM genome through chromosomal recombination. While this recombination process was essential for successful integration, it carried a potential risk of introducing linked drag. However, the MGM strain harbored the *ZmAA1* gene, controlled by the *PG47* promoter, a pollen-specific element (Figure 1A). This gene functions as a gametophytic energy-depleting factor, specifically targeting starch hydrolysis in pollen carrying the MGM cassette. Importantly, MGM can only be transmitted through female gametophytes, resulting in hemizygous inheritance in MGM transgenic plants. This mechanism effectively prevents the chromosomal-linked drag segments present in Z372-MGM from being transmitted to Z372-GMS through male gametes (Figure 6). As a result, the Z372 GMS plants ultimately derived from this approach are free of linked drag.

Our findings hold significant implications for maize breeding, particularly for recalcitrant inbred lines where traditional transformation approaches are less efficient. The use of CRISPR/Cas9-mediated gene editing combined with backcross breeding represents a robust and efficient method for generating GMS and MGM lines, paving the way for more efficient hybrid seed production. Furthermore, the non-transgenic nature of the GMS lines we generated addresses concerns related to transgene integration and opens new avenues for developing commercially viable GMS lines in diverse maize varieties.

This research underscores the potential of gene editing technologies to revolutionize crop breeding, accelerating the development of improved cultivars with enhanced traits for sustainable agriculture. Future research should focus on extending the application of this approach to a broader range of maize varieties and exploring its potential in other plant species. By optimizing gene editing strategies and breeding methods, we can further enhance the efficiency and efficacy of developing GMS and MGM lines, ultimately contributing to a more sustainable and productive agricultural landscape.

## 4. Materials and Methods

### 4.1. DNA Extraction from Maize Leaf Tissue Using the CTAB Method

Plant genomic DNA was extracted from the leaves of F1, BC1F1, BC2F1, and BC2F2 generation plants using the CTAB method as described by Doyle et al. [17]. Fresh corn leaf tissue was placed in a 2 mL centrifuge tube containing a steel ball, rapidly frozen in liquid nitrogen, and ground to a powder using a grinding apparatus. Preheated CTAB extraction buffer (0.05 M CTAB, 1.4 M NaCl, 0.1 M Tris-HCl, 0.02 M EDTA, adjusted to 1 L with ddH_2_O) at 60 °C was added, and the mixture was thoroughly vortexed. The tubes were incubated in a 60 °C water bath for 30 min to lyse the tissue. Following incubation, chloroform/isoamyl alcohol was added, the mixture was vortexed, and allowed to stand for 5 min to facilitate phase separation. After centrifugation, the supernatant was collected, and DNA was precipitated with isopropanol. The DNA pellet was washed with 70% ethanol, air-dried, and resuspended in an appropriate volume of deionized water. The extracted DNA was stored at −20 °C for subsequent use in the detection of the *Cas9* gene and mutation analysis of the *MS26* gene.

### 4.2. Detection of Genetically Modified Component

Following established protocols [16], Z372 MGM plants were screened using DsRed fluorescence as a reliable indicator to select MGM-positive kernels and exclude GMS-negative kernels. To identify and screen for the T-DNA components of CRISPR-Cas9 in both MGM and GMS materials, PCR reactions were performed using the Cas9-F and Cas9-R primers. Additionally, mutations in the ms26 gene target were identified in both MGM and GMS samples using the MS26-F and MS26-R primers (Supplementary Table S4). PCR cycling conditions were as follows: initial denaturation at 95 °C for 5 min, followed by 35 cycles of denaturation at 95 °C for 15 s, annealing at 60 °C for 30 s, and extension at 72 °C for 30 s, concluding with a final extension step of 5 min.

### 4.3. The Identification of MGM Transgenic Copy Number

In this study, we followed our previously established protocols [14] to carry out the experimental procedures. Initially, ten BC2F2 lines, ranging from Z372-MGM1 to Z372-MGM10, were subjected to genomic DNA extraction. The extracted DNA was then digested using the *Hind*III enzyme (New England Biolabs, MA, USA). Next, we added pre-designed primers and probes, as detailed in Supplementary Table S4, to the reaction system. After setting up the reaction, we mixed Bio-Rad droplet generation oil (BioRad, Pleasanton, CA, USA) with the reaction mixture and loaded it into a droplet generator cartridge (Bio-Rad, Pleasanton, CA, USA) to create droplets. These droplets were carefully transferred from the cartridge to a 96-well PCR plate (Bio-Rad, Pleasanton, CA, USA) and securely sealed with a pierceable foil seal (Bio-Rad, Pleasanton, CA, USA). The sealed plate was then placed in a thermal cycler to carry out the PCR reactions. Once the thermal cycling was completed, the plate was transferred to a QX200™ droplet reader (Bio-Rad, Pleasanton, CA, USA) for analysis. Using the default settings and a threshold for positive and negative droplet discrimination, we analyzed the droplet counts. Finally, we employed Bio-Rad QuantaSoft™ software (v1.6.6.0320) to generate measurements of the transgenic copy number.

### 4.4. Copy Number Calculating

The copy number calculation method, as utilized by Hindson et al. [18] employing droplet digital PCR (ddPCR), was referenced to determine the copy numbers of ten samples from the BC2F2 generation, Z372-MGM1 to MGM10. Copy number analysis (CN), which quantifies the copy number of a target gene relative to an invariant reference gene, was conducted using dual-target and reference assays to measure MGM T-DNA and *ZmADH1* genes. Within QuantaSoft™ software, the copy number was ascertained by calculating the ratio of the target molecule concentration to the reference molecule concentration, multiplied by the copy number of the reference species in the genome, typically two. The formula for calculation is expressed as CN = A/B × NB, where A represents the concentration of the target species, B represents the concentration of the reference species, and NB denotes the copy number of the reference locus in the genome.

### 4.5. Whole-Genome SNP Genotyping

Gene chip detection was performed following the methods described by Guo et al. [19], and data analysis was conducted using a multi-step approach. The gene chip detection was conducted by Shijiazhuang Molbreeding Biotechnology Co., Ltd., and completed using DNBSEQ-T7 gene sequencer (Complete Genomics, San Jose, CA, USA) Initial data processing involved background marker filtering, selecting only homozygous and inconsistent loci between the parental lines. Marker integrity filtering was then performed, extracting the SNP with the lowest missing proportion in each mSNP region as the representative SNP marker for that region. Genotype correction and imputation were conducted using SMOOTH software, which leverages the principle that recombination events tend to occur in specific fragments. The background recovery rate was calculated using the filtered markers. Finally, backcross-introgressed segments were identified using a customized Perl script, which leveraged the corrected genotypes to accurately determine the introgressed segments.

### 4.6. Whole-Genome SNP Analysis and Genotype Correction Using SMOOTH Statistical Method

Whole-genome SNP analysis was conducted using the SMOOTH statistical method [20], which facilitated the correction of potential errors in progeny genotypes at specific loci and the imputation of partially missing sites. Genotype markers that were homozygous and consistent between the recurrent parent and progeny were designated as ‘A’, while those that were homozygous and inconsistent were labeled ‘B’. Markers that were homozygous in the recurrent parent and heterozygous in the progeny, with at least one genotype derived from the parent’s homozygosity, were marked as ‘H’. Genotype markers with missing data in either the recurrent parent or the progeny were denoted as ‘N’, resulting in a corrected ABH file. Consequently, the homozygosity rate of the parent Z372 in the whole genome, represented by the number of ‘A’ markers, was calculated as the ratio of ‘A’ markers to the total number of valid SNP markers present in both the recurrent parent and progeny. Similarly, the homozygosity rate of the parent ZC01 was determined by the count of ‘B’ markers against the total valid SNP markers. The heterozygosity rate in the whole genome of both parents was assessed by the number of ‘H’ markers divided by the total valid SNP markers present.

### 4.7. Pollens Staining with Iodine–Potassium Iodide (KI/I_2_)

To visualize the stamens and pollen grains, we adhered to the staining protocol detailed by Hunt et al. [21] utilizing an iodine–potassium iodide (KI/I_2_) solution. A mature anther was placed on a microscope slide, and 1–2 drops of a 100-fold diluted Lugol’s solution (10% [*w*/*v*] potassium iodide, 5% [*w*/*v*] iodine) were applied for staining. Using forceps, the anther was gently pressed to facilitate the release of pollen grains and their contact with the staining solution. A cover slip was carefully placed on one side of the droplet, and the preparation was allowed to stand for 1–2 min before examination under an optical microscope. Fresh anthers were fixed with FAA fixative (formalin–acetic acid–ethanol) and then directly stained with Lugol’s solution for 5 min before observation.

### 4.8. Imaging and Fluorescence Observation

We observed the stained pollen grains through a Nikon stereomicroscope (SMZ1500, Japan) at a 10× magnification. Additionally, we examined freshly treated anthers under a Zeiss microscope (LSM700, Oberkohen, Germany) in bright view mode, utilizing a 10 × 4 magnification. For the identification of MGM maize ears and kernels, we employed a fluorescent flashlight (DFP-1; Electron Microscopy Sciences, Hatfield, PA, USA) equipped with a 550 nm excitation wavelength and DsRed-specific filter glasses, following the protocol described by Dong et al. [13]. Furthermore, to visualize the longitudinal sections of MGM-positive seeds, we used the Nikon stereomicroscope (SMZ1500) again, this time with the same 550 nm excitation wavelength and DsRed-specific filter glasses, but at a 10 × 0.75 magnification.

### 4.9. Chi-Square Analysis

To ascertain the ratio of viable to non-viable pollen in the anthers of Z372-MGM2, the chi-square (χ^2^) test, as reviewed by Franke et al. [22], was employed to measure the deviation between observed and expected values of the sample. Observations of viable and non-viable pollen counts were taken three times, and the mean values were calculated. Using SPSS, the goodness of fit to a 1:1 ratio was computed, yielding a chi-square value of 0.618. Reference to the chi-square table revealed that, with one degree of freedom, the critical value for significance at the 0.05 level is 3.84. Consequently, it was determined that the observed ratio of viable to non-viable pollen in Z372-MGM2 anthers aligns with the expected 1:1 ratio.

### 4.10. Field Experiment and Trait Measurements

The experimental setup consisted of plots measuring 4 m in length and 1.2 m in width, with three replicated experiments carried out. To mitigate the potential impact of edge effects on our findings, only plants situated in the center of each plot were selected for sampling. Once the plants reached maturity, meticulous records were taken for each plant, including plant height and the number of leaves. Following harvesting, kernel row number and kernels per row were carefully documented as well.

### 4.11. Planting and Management of Materials across Generations

The experiment encompassed the cultivation and pollination of materials across five generations. In the initial generation, designated as the parental generation, we planted 18 individuals of the ZC01-3A-7 and Z372 materials in separate rows. From these, a single healthy individual of each, with ZC01-3A-7 serving as the female parent and Z372 as the male parent, was selected for cross-pollination, yielding F1 seeds. During the F1 generation cultivation, a row of Z372 was planted alongside 20 seeds of the (ZC01-3A-7 × Z372) F1 cross. Post-emergence, DNA was extracted from leaf samples for genotyping MGM, Cas9, and MS26 genes. Based on our experimental objectives, three F1 individuals, namely (ZC01-3A-7 × Z372)-4, (ZC01-3A-7 × Z372)-7, and (ZC01-3A-7 × Z372)-12, were chosen as female parents and crossed with Z372 to produce the BC1F1 generation. In the BC1F1 generation, a row of Z372 was again planted, and 93 BC1F1 seeds were sown. After germination, leaf DNA was extracted and genotyped for MGM, Cas9, and MS26 genes. Twenty-three individuals (listed in Supplementary Table S1) were selected as female parents and crossed with Z372 to yield the BC2F1 generation. For the BC2F1 generation, a row of Z372 was planted, and 94 BC2F1 seeds were sown. Upon emergence, leaf DNA was extracted and genotyped for MGM, Cas9, and MS26 genes. Four individuals, Z372/7/2, Z372/17/2, Z372/17/3, and Z372/64/1, were selected for self-pollination to generate the BC2F2 generation. In the BC2F2 generation, 94 individuals were sown. Post-emergence, leaf DNA was extracted and genotyped for MGM, Cas9, and MS26 genes, as well as genetic background. Subsequently, individuals identified as Z372-MGM and Z372-GMS were selected for further study.

## 5. Conclusions

In conclusion, we have developed an efficient and reliable breeding strategy for generating genic male sterility (GMS) and maintainer lines in maize. Our approach leverages the in vivo activity of CRISPR/Cas9, coupled with backcross breeding and gene chip detection technology, to achieve high background recovery rates as early as the BC2F2 generation. Importantly, the generated GMS lines are transgene-free, eliminating linkage drag and making them ideal for commercial hybrid seed production. This research demonstrates the significant potential of gene editing technology for the rapid and efficient development of GMS and MGM lines, paving the way for its broader application in diverse maize varieties and other plant species.

## 6. Patents

A Chinese patent (no. ZL201710223233.1) corresponding to this study has been authorized.

## Figures and Tables

**Table 1:** Generation of *ms26ΔE5* mutations using in vivo Cas9 activity among MGM and GMS progeny of the Z372 line.

Sample Name	Sequence (5′ to 3′) ^1^
Z372	CCTCCGCGATGCAACGCATATGTGGC-//-CCGCTTAATCCACGACAAATAACG
ZC01-3A-7	CCTCCGGTCCGCGA---------------------------//-------------AATCCACGACAAATAACG
Z372-MGM1	CCTCCGGTCCG-GA---------------------------//--------------AATCCACGACAAATAACG
Z372-MGM2	CCTCCGGTCCG-GA---------------------------//--------------AATCCACGACAAATAACG
Z372-MGM3	CCTCCGGTCCGCGA--------------------------//-----------------------------GACAAATAACG
Z372-MGM4	CCTCCGGTCCGCGA-------------------------//------------------------------GACAAATAACG
Z372-MGM5	CCT--------------------------------------------------//-----------------------------GACAAATAACG
Z372-MGM6	CCT------------------------------------------------//-------------------------------GACAAATAACG
Z372-MGM7	CCT------------------------------------------------//-------------------------------GACAAATAACG
Z372-MGM8	CCTCCGGTCCGCGA-----------------------//----------------------CCACGACAAATAACG
Z372-MGM9	CCTCCGGTCCGCGA-----------------------//----------------------CCACGACAAATAACG
Z372-MGM10	CCTCCGGTCCGCGA-----------------------//----------------------CCACGACAAATAACG
Z372-GMS1	CCTCCGGTCCG-GA------------------------//-----------------AATCCACGACAAATAACG
Z372-GMS2	CCTCCGGTCCG-GA------------------------//-----------------AATCCACGACAAATAACG
Z372-GMS3	CCTCCGGTCCGCGA-----------------------//-------------------------------GACAAATAACG
Z372-GMS4	CCTCCGGTCCGCGA------------------------//------------------------------GACAAATAACG
Z372-GMS5	CCTCCGGTCCGCGA------------------------//------------------------------GACAAATAACG
Z372-GMS6	CCT------------------------------------------------//-------------------------------GACAAATAACG
Z372-GMS7	CCT-----------------------------------------------//--------------------------------GACAAATAACG
Z372-GMS8	CCT-----------------------------------------------//--------------------------------GACAAATAACG
Z372-GMS9	CCT------------------------------------------------//--------------------------------GACAAATAACG
Z372-GMS10	CCT------------------------------------------------//--------------------------------GACAAATAACG

^1^ Detailed genotype information is listed in Supplementary Table S2.

**Table 2:** Whole-genome SNP analysis of background restoration rate in Z372-MGM and Z372-GMS.

Sample Name	Total SNPs	Z372 SNPs (%)	ZC01 SNPs (%)	Heterozygous SNPs (%)	Background Restoration Rate (%)
Z372-MGM1	25,727	89.67	0.23	10.09	94.71
Z372-MGM2	25,728	92.89	0.23	6.88	96.32
Z372-MGM3	25,705	88.08	0.41	11.51	93.82
Z372-MGM4	25,723	89.90	0.27	9.82	94.80
Z372-MGM5	25,722	89.01	0.26	10.73	94.36
Z372-MGM6	25,719	91.64	0.28	8.08	95.66
Z372-MGM7	25,724	92.89	0.23	6.88	96.32
Z372-MGM8	25,725	92.24	0.19	7.57	96.02
Z372-MGM9	25,727	88.22	0.23	11.54	93.99
Z372-MGM10	25,730	92.03	0.20	7.77	95.92
Z372-GMS1	25,729	91.29	0.25	8.46	95.52
Z372-GMS2	25,726	90.43	0.30	9.27	95.06
Z372-GMS3	25,728	92.25	0.29	7.46	95.98
Z372-GMS4	25,725	93.04	0.43	6.53	96.30
Z372-GMS5	25,728	96.04	0.24	3.72	97.89
Z372-GMS6	25,730	97.33	0.21	2.46	98.56
Z372-GMS7	25,730	90.43	0.22	9.35	95.10
Z372-GMS8	25,730	97.64	0.17	2.19	98.74
Z372-GMS9	25,730	96.23	0.17	3.60	98.03
Z372-GMS10	25,727	95.76	0.14	4.10	97.80

**Table 3:** The chi-square test was performed to analyze the proportions of fertile and sterile pollen in Z372-MGM2.

Pollen	Observed	Expected	χ^2^ (1:1)	χ^2^ 0.05, 1 (1:1)
Yellow pollens	1480	1518	0.618	3.84
Purple pollens	1556	1518		
Total number	3036	3036		

## Data Availability

The data are contained within the manuscript and Supplementary Materials.

## Supplementary Materials

The following supporting information can be downloaded at https://www.mdpi.com/article/10.3390/ijms25115832/s1.
